# Our core values will endure

**DOI:** 10.1080/02813432.2020.1842676

**Published:** 2020-12-30

**Authors:** Johann A. Sigurdsson, Anders Beich, Anna Stavdal

**Affiliations:** a Chair of the Nordic Federation of General Practice, GP, Professor emeritus, Development Centre for Primary Health Care, Iceland; Department of Public Health and Nursing; General Practice Research Unit, Norwegian University of Science and Technology, Trondheim, Norway; b Chair of the Danish College of General Practice, GP, Copenhagen, Denmark; c WONCA World, GP, President Elect, The Norwegian College of General Practice, Oslo, Norway

In order to define what our discipline stands for, and what we are fighting for on its behalf, the Nordic Colleges of General Practice has formulated a statement of the Core Values *and* Principles of Nordic General Practice/Family Medicine, published in this issue.

## Why now? Societal trends and new challenges

Family Medicine was established as a specialty in its own right in some vanguard Western countries during the 1960s and 70s. Equal access to care, continuity of care, comprehensive care, and solidarity, reflected the political ethos of the times [1]. Its ideology was in large part encapsulated in this quote from Ian McWhinney:

When taken together, the principles of Family Medicine represent a worldview – a system of values and approaches to problems – that is identifiably different from that of other disciplines [2]

During the following decades, societal trends have impacted both the context and the standards of care, that is, workload, tasks and organization within healthcare in general, and in General Practice in particular. Some of these trends have been described in this journal [3–10].

Many of the trends and challenges are apparently interlinked: changes regarding economic constraints and incentives, specialisation and fragmentation of care, ever-increasing mobility among patients and professionals, digitalisation, as well as a tendency toward over-medicalisation within many specialties – all developed within a context of greater commercialisation and a steepening social gradient. Furthermore, opportunistic ‘digital doctoring’ companies have emerged, alongside a growing interest among health authorities, investors, and researchers in monitoring consultations and analysing ‘Big Data’. Problems related to ‘too much medical overactivity’, overdiagnosis, and overtreatment are on the rise [10,11].

## The importance of core values

Carefully chosen core values serve as guiding principles. These become particularly important when the stakes are high and alternative courses of action exist. Identifying essential values and then acting in accordance with them lays the groundwork for transparency, and for trust to develop [12–14]. Which of the core values and principles formulated during the 70s are still valid and applicable, given the wide range of challenges that have emerged? It is important to note here that WONCA Europe did formulate a definition of general practice in 2002 [15], highlighting essential ideological concepts and core professional competencies for GPs. However, the WONCA definition did not explicitly emphasise the relationship between viable working principles and moral values, as we do here.

## The Nordic core values and principles

In 2017, the Nordic Federation of General Practice (NFGP), the collaborative union of the five Nordic Colleges of General Practice, decided first to re-think and revise their vision and mission statements, and then to formulate their core values and guiding principles.

**Figure 1.**
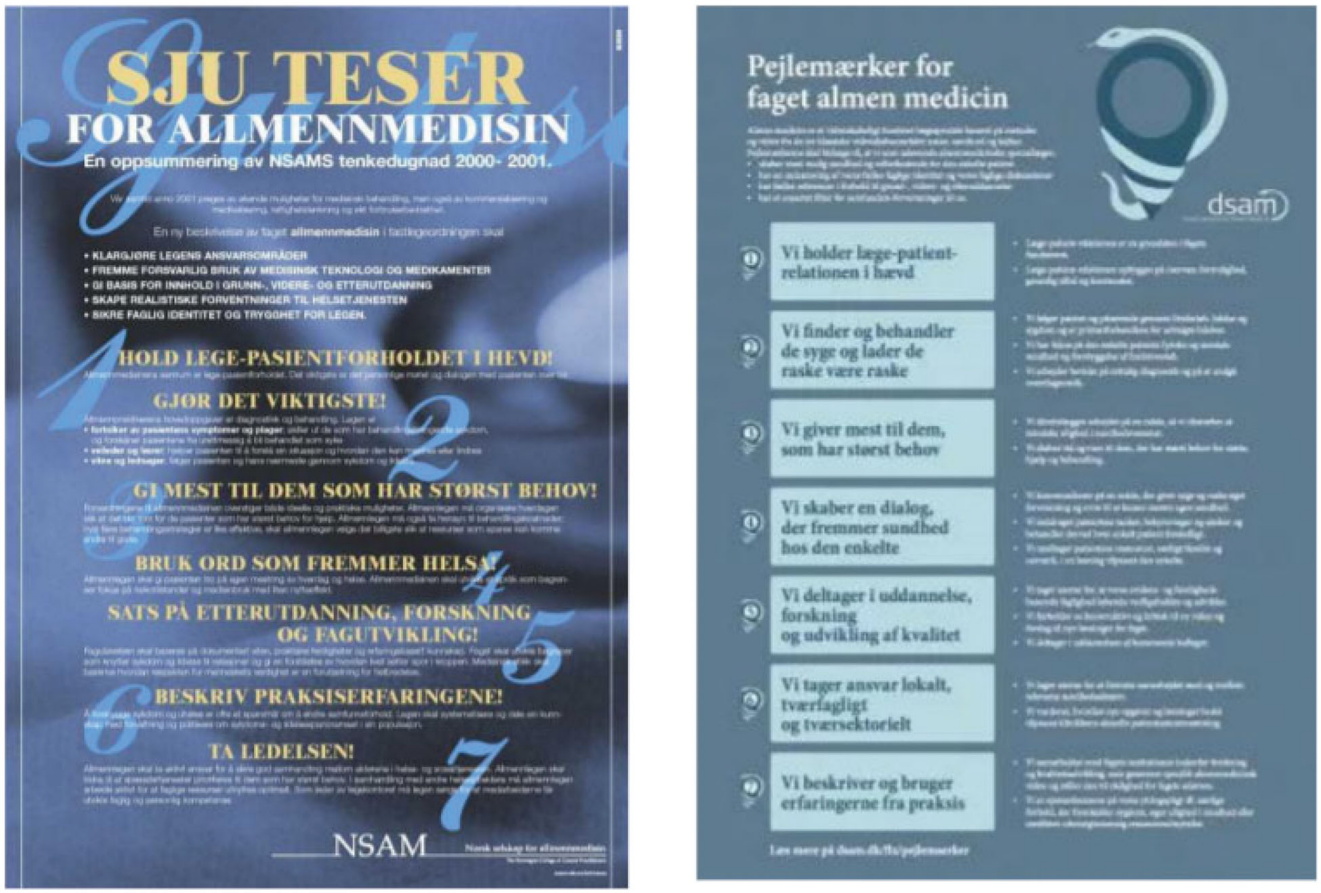
Poster versions of the Norwegian ‘Sju teser’ (2001) and the Danish ‘Pejlemaerker’ (2016) statements.

Discussions of that sort had a long history among several of the Nordic countries. The experiences, conversations, and debates of the late nineties resulted in the Norwegian College of General Practice (NSAM, later NFA), chaired by one of the authors here (AS), taking the lead to draw up ‘Sju teser’ (‘Seven principles of good medical practice for General Practitioners’) These ‘Sju teser’ were published in Norwegian in 2001, formatted and distributed in the form of an eye-catching poster [16,17] (Figure 1).

Fifteen years later, the Danish College of General Practice (DSAM), led by another of the authors (AB), decided to analyse data from an extensive vision process, involving hundreds of Danish GPs, in which they had shared their views on the core values, principles and purposes of General Practice. In 2016, based on this input and strongly inspired by the ‘Sju teser’ of 2001, DSAM formulated their own, updated, version of the professional principles and values, called ‘Pejlemaerker for faget almen medicin’ (‘Guideposts for the Profession of General Practice Medicine’) [18] (Figure 1). The history of these projects can be read here: https://www.nfgp.org/files/34/status_report_working_group_on_core_values_and_principles.pdf.

In the above-mentioned 2017 revision process, it proved unrealistic to simply merge and then translate the Norwegian and Danish versions into an English version that satisfied everyone. We needed first to scrutinize and debate the unexpectedly divergent associations and cultural implications that the Danish, Norwegian, and English words and concepts carried. And again, hundreds of Nordic GPs were involved, participating in workshops and digital exchanges. The diversity of opinions and suggestions that emerged was enriching and greatly appreciated. The English rendering of the Nordic GPs shared understanding of the Nordic statement was agreed to at last, as it appears in this issue. We find it particularly noteworthy that, despite two decades of applying and debating these perspectives, their actual contents – our values and principles – have hardly changed.

## Research supporting the core values

A substantial body of literature and research supports the relevance and validity of core values and principles of General Practice [5,9,10,19]. Nonetheless, more work is needed. For example, it has been pointed out that many of the research studies regarding values have soft endpoints regarding moral intent, which are difficult to define and, consequently, difficult to validate. Most of the studies so far have focused on concepts such as ‘generalist/comprehensive care’ and the doctor-patient relationship [20].

Through ground-breaking research using morbidity and mortality as hard endpoints, Barbara Starfield and her co-workers were able to document better outcomes in regions/countries with a higher number of primary care physicians, compared to those with a lower number [21,22].

Relationship-based care is one of the cornerstones of general practice [5,8,9]. Yet, due to societal trends in many countries, the organization of contemporary healthcare systems threatens the doctor-patient relationship mode of care. It becomes even more important to stress such results as those of a systematic review study recently published by Pereira Gray and co-workers, showing that increased continuity of care by doctors was associated with lowered mortality rates [23].

## Values and standards

To put core values into perspective, we need to clarify the distinction between values and standards. Values have a fundamental, moral intention and help us move toward achieving our vision, particularly in times of tension and dispute. Standards, on the other hand, are more pragmatic. They may vary and change to some extent, even among countries where general practice is considered to have a stronghold. For example, in our Nordic model, unlike those of many other regions, GPs not only take care of patients but also play a crucial role in the management of social and welfare services. Meanwhile, both the recently published values statement from Scotland, ‘The Edinburg Consensus Statement’ [14,24], and the ‘Position paper. Core Values of General Practice/Family Medicine’ from the Dutch College of General Practice [25], show remarkable similarities to our *Core Values and Principles of Nordic General Practice/Family Medicine.* This would seem to indicate that, despite differences in training routines and organisation, our shared core values are what constitute the fundamental and defining basis of our profession. Thus, while standards may vary, our core values and principles should be able to endure and unite us.

Ideally, standards of care will be informed by the best evidence available at any given time. However, both organisational principles and clinical priorities are repeatedly challenged by stakeholders outside of our discipline, as outlined above. In such situations, a common set of Core Values and Principles can both motivate and mobilize us, as advocates for our discipline.

We hope the Nordic Statement of Core Values and Principles can become a beneficial resource, not only for the primary care community but for politicians and stakeholders throughout the entire healthcare system, for researchers and teachers in pre- and postgraduate education, for everyone involved in professional development – and finally, for our patients, the citizens of the Nordic countries.
